# Vaccine safety beliefs in the state of Alaska

**DOI:** 10.1016/j.puhip.2024.100482

**Published:** 2024-02-27

**Authors:** R. David Parker, Jennifer A. Meyer

**Affiliations:** a Yukon Kuskokwim Health Corporation, Bethel, AK, USA; b University of Alaska Anchorage, Division of Population Health Sciences, Anchorage, AK, USA

**Keywords:** Vaccine hesitancy, Vaccine confidence, Public health, Infectious diseases, Epidemiology

## Abstract

**Objectives:**

Identifying the key factors associated with vaccine hesitancy remains a challenge as has been highlighted throughout the COVID-19 vaccine roll out and pandemic. The aim of this study was to determine characteristics associated with vaccine safety and compare perceived safety by vaccine. Our hypothesis is that vaccine safety perception will vary by vaccine with COVID-19 as ranked lowest for safety.

**Study design:**

Cross sectional.

**Methods:**

A statewide sample (n = 1024) responded to an online 28-point questionnaire via anonymous linked invitation.

**Results:**

Among the eight vaccines assessed, COVID-19 had the lowest perceived safety (53.13%) followed by human papillomavirus HPV (63.38%). A binomial logistic regression assessed COVID-19 vaccine safety beliefs (safe v not safe) finding age, political orientation, and perceived safety of certain vaccines as statistically significant. As age increased by year, vaccine safety beliefs increased. Persons who identified as conservative demonstrated less belief in vaccine safety than all other groups. Among persons who did not perceive the COVID-19 vaccine as safe, 65.8% believed chicken pox was safe, 63.3% and 61.1% perceived hepatitis A& B were safe.

**Conclusions:**

These findings demonstrate that vaccine safety beliefs differ by vaccine and that persons who do not believe in the safety of the COVID-19 are not exclusively against all vaccines. Understanding factors that increase vaccine safety by vaccine could assist in developing an intervention which could increase belief in safety for all vaccines.

## 1 Introduction

In the United States, vaccine safety has been a priority for over 120 years beginning in 1902 with the Biologics Control Act, followed by the Food and Drug Act in 1906. These efforts led to the creation of the Food and Drug Administration (FDA), the organization responsible for monitoring medication and food supply safety in the United States [1]. Vaccine development is like other pharmaceutical development, which includes extensive testing for efficacy, safety, tolerability, and post-approval surveillance for side effects and safety [[2], [3], [4]]. Vaccine reactions and side effects are rare, and the evidence indicates the efficacy of approved vaccines in reducing morbidity, mortality, and healthcare utilization [[5], [6], [7]].

Despite government safety regulations and significant resources dedicated to vaccine development over many years, the antivaccine movement grew in response to the publication of a since retracted manuscript which linked autism with the measles, mumps, and rubella (MMR) vaccine in the 1980s [8]. Thousands of studies have refuted these findings and have assessed factors associated with vaccine hesitancy, including political party, political ideology, education, race, sex, age, income, and similar demographics [5,9,10]. During the recent COVID-19 pandemic, the global response was highly politicized, based in part on restrictions, lockdowns, accelerated vaccine development, and allocation of resources. The COVID-19 vaccine provides an opportunity to measure vaccine hesitance in the context of vaccines approved outside of a pandemic. COVID-19 vaccine hesitancy has been presented as a concept closely tied to anti-vaccine sentiment in many studies [11]. While persons against vaccine would likely oppose the COVID-19 vaccine, it is possible that some people who are hesitant about the COVID-19 vaccine are not against vaccines, but rather, may have been overwhelmed by the ever-changing information available on a novel, emerging pathogen.

Our study measured vaccine safety beliefs among a population of persons living in a rural and remote state. Our hypothesis is that persons will have different perceptions of vaccine safety with the least confidence in the COVID-19 vaccine. Determining if differences in hesitance exists between vaccines, we can better explore pathways to increase vaccine uptake.

## 2 Methods

This project was approved by the institutional review board as exempt. A 28-question survey created for this study was informed by previous work on vaccine hesitancy and was stored in Qualtrics [9,12,13]. An a priori power analysis to detect a 10% effect size difference between groups for a multiple linear regression with eight predictors would require a sample size of 299 persons. Given the state's diversity, we increased our sample size (n = 1000). The sampling was completed in partnership with an established company who maintains panels of people interested in participating in research. The company distributed links to the research team developed survey in December 2022. Persons aged at least 18 years of age, who were current Alaska residents, and who could complete the survey in English were recruited. A system was developed so the recruitment company did not have access to responses and the research team did not have access to participant identifying information. After consenting to participate, respondents completed the survey.

Personally identifying information, including zip code, IP address, latitude, and longitude were collected via Qualtrics and removed from the data once the responses were mapped to the borough level. Alaska is unique in that the state has 19 boroughs (2 urban, 17 rural) and 10 US Census Bureau Designated places. Given the distinct way Alaska's boroughs are mapped and categorized, zip code collection allowed a more precise determination of a participant location. Data were downloaded from Qualtrics into csv format for analyses completed in R 4.1.0 [14]. The survey collected data on demographics and vaccine perception and recommendations. Demographic questions included: sex (male, female, other, decline), year of birth, primary race (Alaska Native/American Indian, Asian or Pacific Islander, Black, White, Other, Decline), highest level of education (less than high school, high school diploma or GED, technical training certificate, some college, associate degree, bachelor degree, graduate degree, decline), current employment status, select all (full time, part time, not employed in the paid workforce, retired, disabled, decline), type of health insurance or coverage, select all (Indian Health service, Medicaid, Medicare, Private Health Insurance, Veteran's Benefits, uninsured, decline), home zip code, do you consider yourself a religious person (yes, no, decline), which best describes your political views (conservative, liberal, moderate, not political, other, decline to answer), and were you born in Alaska (yes, no, decline). Political ideologies of “other” and “decline to answer” were included in case of differences with the reference group. Bivariate analyses between the demographics and the outcome of interest determined those which had a statistically significant relationship.

The two primary variables, vaccine safety and vaccine threat were operationalized as follows. Vaccine safety was assessed using a Likert type scale and the level of agreement with the statement “All vaccines are safe. Choices included “Strongly Agree”, “Somewhat agree”, “Neither agree nor disagree”, “Somewhat disagree” and “Strongly disagree”. If a response other than “strongly agree” (that all vaccines are safe), a list of vaccines were presented for the respondent to identify “Which vaccines are safe?)”. This vaccine list included: “Chicken pox”, “COVID”, “Flu”, Hepatitis A, Hepatitis B, “HPV”, “Pneumonia”, and “Shingles. Perceived vaccine threat was assessed using a Likert type scale and the level of agreement with the statement “Vaccines contain dangerous chemicals”. Choices included “Strongly Agree”, “Somewhat agree”, “Neither agree nor disagree”, “Somewhat disagree” and “Strongly disagree”. A cumulative covariate score was created to enable assessment of vaccine safety in relation to COVID-19 vaccine safety and was the number of vaccines identified as safe, from the list chicken pox, hepatitis A, hepatitis B, human papilloma virus (HPV), influenza, pneumonia, and shingles.

To determine the statistical significance of individual variables, their relationship with the outcome variable was using the most appropriate bivariate analyses (i.e. Fisher's Exact Test, Chi Square, T-test). For statistical modeling to identify the most relevant variables for inclusion in a binary logistic regression, a backwards, stepwise method selected variables. Variable removal based on the statistical significance of the 95% confidence interval and the p-value. At each model iteration, model fit assessed by the Akaike Information Criterion (AIC) and the pseudo-R^2^ to identify characteristics associated with COVID-19 vaccine safety. The initial regression model included the binary outcome variable of COVID-19 vaccine safety (not safe, safe) and the covariates identified as statistically significant which were age, sex, race, education (less than high school, high school, some college, technical school certificate, bachelor's degree, graduate degree), political ideology (conservative, liberal, moderate, decline to answer, and other), and the perceived vaccine safety cumulative score.

## 3 Results

A total of 1500 individualized links were sent to potential respondents, yielding a 69.27% response rate (n = 1039). Of these respondents, 15 were removed from analysis for either not completing the consent, not indicating they were a resident in the state, and/or not meeting the minimum age of 18 years for a total sample size of 1024. More than half of respondents (52.9%, n = 542) were women, held a bachelor's degree (23.5%, n = 241), primarily white (78.1%, n = 800) and Alaska Native (11.6%, n = 119), almost half (47.9%, n = 490) were employed at least full-time. The primary healthcare coverage identified was private health insurance at 61.7% (n = 632). Almost half of the respondents (47.4%, n = 485) report being religious and approximately one-third (35.3%, n = 361) were born in Alaska. Almost one-third of respondents (30%, n = 307) identified as politically conservative, 29.8% (n = 305) were moderate, 21.2% (n = 217) were liberal, 9.8% (m = 100) as ‘not political’ and 5.2% (n = 53) as ‘other.’ Full demographics are reported in Table 1.

**Table 1:** Demographic overview of participants (n = 1024).

Variable	n (%)
**Sex**	
Male	466 (46.5%)
Female	530 (52.9%)
**Age**	49.0 years median, 48.81 mean
**Race**	
Alaska Native/American Indian	116 (11.6%)
Asian or Pacific Islander	25 (2.5%)
Black	10 (1.0%)
Other	40 (4.0%)
White	781 (77.9%)
**Political Orientation**	
Conservative	302 (30.1%)
Liberal	215 (21.5%)
Moderate	295 (29.4%)
Not Political	99 (9.9%)
Other	52 (5.2%)
**Highest Education**	
Less than high school	10 (1.0%)
High School Diploma or Equivalent	143 (14.3%)
Some College	291 (29.0%)
Technical Training Certificate	61 (6.1%)
Bachelor's Degree	236 (23.6%)
Graduate Degree	159 (15.9%)
**Employment Status**	
Full Time	480 (47.9%)
Part Time	145 (14.5%)
Retired	250 (24.4%)
Disabled	17 (1.6%)
Not in the Paid Workforce	120 (12.0%)
**Health Insurance**	
Private Health Insurance	621 (62.0%)
Medicare	226 (22.6%)
Medicaid	130 (13.0%)
Indian Health Service	62 (6.2%)
Veteran's Benefits	60 (6.0%)
Uninsured	62 (6.2%)
**Born in the State of Alaska**	
Yes	353 (35.2%)
No	648 (64.7%)

Overall, 20.3% (n = 208) of persons strongly agreed that all vaccines were safe, 35.6% (n = 365) somewhat agree that all vaccines are safe, 11.62% (n = 119) neither agree nor disagree that all vaccines are safe, 16.4% (n = 168) somewhat disagree that all vaccines are safe, and 15.9% (n = 163) strongly disagree that all vaccines are safe. Among participants who did not “strongly agree” that all vaccines were safe (n = 815), the COVID-19 vaccine had the lowest perceived safety at 41.20% (n = 336). Table 2 provides an overview of perceived vaccine safety. The COVID-19 vaccine is perceived as the least safe among all three groups, however, among persons who do not perceive the COVID-19 vaccine as safe, the perceived safety of other vaccines is lowest.

**Table 2:** Perceived vaccine safety.

Vaccine	Believe Vaccine is Safe		
	Total Sample (n = 1024)	Not all vaccines are safe, but this vaccine is safe. n = 815 (79.59%)	COVID-19 vaccine is not safe, but this vaccine is safe. n = 479 (46.78 %)
Chicken Pox	833 (81.35%)	625 (76.72%)	315 (65.8%)
COVID-19	544 (53.13%)	336 (41.20%)	–
Hepatitis A	776 (75.78%)	568 (69.73%)	294 (61.1%)
Hepatitis B	798 (77.64%)	587 (72.06%)	303 (63.3%)
Human Papilloma (HPV)	649 (63.38%)	441 (54.04%)	181 (37.8%)
Influenza	731 (71.39%)	523 (64.22%)	207 (43.2%)
Pneumonia	729 (71.19%)	521 (63.97%)	229 (47.8%)
Shingles	780 (76.17%)	572 (70.22%)	259 (54.1%)

Using a backwards stepwise regression with model fit assessed by the Akaike Information Criterion (AIC) and the pseudo-R^2^, with each variable removal based on the statistical significance of the 95% confidence interval and the p-value, there were 13 model iterations. The final model had the lowest AIC (1005.6), the highest pseudo-R^2^ (0.30), and was the most parsimonious with three statistically significant variables and COVID-19 vaccine safety. A one-year increase in age increased the perception of COVID-19 vaccine safety by 1.92% (OR 1.02; 95% CI, 1.01, 1.03). The impact of political ideology was measured using conservative ideology as the reference group which had the lowest perceived safety of the COVID-19 vaccine. Persons who declined to provide their political ideology were 3.46 times more likely to perceive the COVID-19 vaccine as safe (95% CI: 1.65, 7.21); liberals were 62.98 times as likely (95% CI: 33.74, 126.67); moderates were 7.21 times as likely (95% CI: 4.91, 10.71); persons identifying as “not political” were 3.90 times as likely (95% CI: 2.24, 6.64); “Other” were 2.91 times as likely (95% CI: 1.47, 5.68).

For persons who did not agree that all vaccines were safe, some individual vaccines were statistically associated with the perception of COVID-19 vaccine safety. Persons who believed the HPV vaccine and the influenza vaccine were safe were 2.65 (95% CI: 1.70, 4.17) times and 3.90 (95% CI: 2.48, 5.89) times as likely to believe the COVID vaccine was safe. Belief that some vaccines were safe was found to decrease the belief in the COVID-19 vaccine safety, including chicken pox at 0.42 (95% CI: 0.26, 0.66) and hepatitis B at 0.36 (95% CI: 0.23, 0.58). This model is presented in Table 3.

**Table 3:** Final logistic regression model: Characteristics associated with COVID-19 vaccine safety (n = 1024).

Characteristics	Estimate	Std. error	z-value	p-value	Odds ratio	95% CI (LL, UL)
Intercept	−2.36	0.36	−6.61	<0.001	0.09	0.05, 0.19
Age (1 year)	0.019	0.01	3.61	<0.001	1.02	1.01, 1.03
Political Ideology^a^						
Declined	1.24	0.37	3.31	<0.001	3.46	1.65, 7.21
Liberal	4.14	0.34	12.31	<0.001	62.98	33.74, 126.67
Moderate	1.98	0.20	9.95	<0.001	7.21	4.91, 10.71
Not Political	1.35	0.28	4.88	<0.001	3.90	2.24, 6.64
Other	1.07	0.34	3.11	<0.001	2.91	1.47, 5.68
Vaccine Safety						
HPV	0.97	0.23	4.27	<0.001	2.65	1.70, 4.17
Influenza	1.33	0.22	6.10	<0.001	3.90	2.49, 5.89
Chicken Pox	−0.88	0.24	−3.69	<0.001	0.42	0.26, 0.66
Hepatitis B	−1.01	0.24	−4.27	<0.001	0.36	0.23, 0.58

The original model included: age, sex, race, education, political ideology, borough of residence, employment status, select all (full time, part time, retired, disabled, not employed), insurance provider, select all (private health insurance, Medicare, Medicaid, Indian Health Service, uninsured, Veteran's Affairs), and perception of vaccine safety (chicken pox, COVID, hepatitis A, hepatitis B, human papilloma virus (HPV), influenza, pneumonia, and shingles).

Model fit: AIC = 1006, Pseudo r^2^ = 0.31.

a Conservative ideology is the reference group.

## 4 Discussion

The investigation supported our hypothesis that the COVID-19 vaccination would have a low perception of safety, as 53.13% (n = 544) of our sample believe the COVID-19 vaccine is safe. The vaccine with the second lowest perceived safety is HPV (63.38%, n = 649). Both vaccines have been met with significant stigma, COVID-19 on the rapid approval process and the HPV vaccine due to its association with sexual activity. Despite the efficacy of the HPV vaccine, nationally, the uptake is at 54% of adolescent women [15]. Safety concerns were reported as the main reason for not using the HPV vaccine series from 13.0% in 2015 to 23.4% in 2018. Data from the Centers for Disease Control (CDC) WONDER demonstrate that between 2020 and 2022, there were 3763 HPV vaccine adverse events reported events, of which 99 were serious adverse events [16]. Our results suggest that vaccines associated with social stigma have low perceived safety. Perhaps public education on the safety of individual vaccines could be a potential intervention but this needs further investigation.

An interesting relationship was found between vaccine safety views for chicken pox and hepatitis B – where persons who identified as conservative were more likely to consider those vaccines as safe even though they do not perceive the COVID-19 vaccine as safe. After reviewing the literature, this could be attributed to the duration of approval for both vaccines combined with a less public, less political stigma. This opens an area for further explanation if we could identify why certain people perceiver some vaccines as safe and not others.

In our multivariable, binomial regression, self-identified conservatives were least likely to believe in COVID-19 vaccine safety, especially persons identifying as liberal. By asking for political ideology, we were able to measure conservatism on a spectrum. This could explain why our findings indicate that all ideologies aside from conservative are more likely to view the COVID-19 as safe, compared to political party or presidential voting choice in 2020 [5,9,17].

Our study has limitations, primarily surrounding our sampling technique. We used an online survey tool sent to people who had already agreed to be potential research participants. Persons who have already agreed to consider research participation may be distinctly different from persons who would not agree and may differ based on some underlying characteristics which enabled them to be contacted by the recruitment organization. An online survey administration limits our access to people with a mobile device or computer and some type of internet connection. Our survey was conducted in English only, which excludes ∼10% of the residents in the State of Alaska. To mitigate some of these concerns, we oversampled. Our sample size calculation indicated that to yield 80% power a sample size of 199 persons was needed to detect moderate differences between groups. Instead, we recruited 1024 people. At baseline, the demographic characteristics appeared equally distributed, so while the limitations on recruitment remain, we believe we reduced some bias which could exist through disproportionate sampling. We measured political ideology in lieu of either “political party” or “who did you vote for in the last presidential election” given that emerging literature indicates people tend to align with ideologies such as conservative, liberal, or moderate differently than party affiliation. A person can be liberal and republican or conservative and democrat [9,10]. An additional possible limitation was that at the time of this project, no validated instrument was identified. We believe this impact to be minimal as our outcome variable was assessed by the level of agreement with the statement “All vaccines are safe.”. A Likert Type Scale provided five potential responses ranging from “strongly agree” to “strongly disagree”. As a single question, a measure of reliability such as Cronbach's Alpha would not be applicable [18].

Further work will be conducted to understand vaccine safety. While COVID-19 vaccine was perceived as the least safe, determining factors associated with vaccine safety by individual vaccine (influenza, chicken pox, HPV) is important. Within our sample, it appears that political ideology and current vaccine acceptance are key indicators for new vaccines and specific vaccines. These findings indicate that if a person does not believe the COVID-19 is safe, this belief does not always translate to other vaccines. This presents a more complex understanding of vaccine hesitancy and suggests that not all persons who do not believe in the safety of the COVID-19 vaccine are against vaccines.

## Ethical approval

This study was approved by the Institutional Review Board as exempt.

## Funding

Funding for this work was provided in part by the State of Alaska 10.13039/100004856Department of Health.

## Declaration of competing interest

The authors declare that they have no known competing financial interests or personal relationships that could have appeared to influence the work reported in this paper.
